# Natural variation in floral nectar proteins of two *Nicotiana attenuata* accessions

**DOI:** 10.1186/1471-2229-13-101

**Published:** 2013-07-13

**Authors:** Pil Joon Seo, Natalie Wielsch, Danny Kessler, Ales Svatos, Chung-Mo Park, Ian T Baldwin, Sang-Gyu Kim

**Affiliations:** 1 Department of Molecular Ecology, Max Planck Institute for Chemical Ecology, Hans-Knöll-Straße 8, Jena, D-07745, Germany; 2 Mass Spectrometry/Proteomics Research Group, Max Planck Institute for Chemical Ecology, Hans-Knöll-Straße 8, Jena, D-07745, Germany; 3 Department of Chemistry, Chonbuk National University, Jeonju, 561-756, Korea; 4 Molecular Signaling Laboratory, Department of Chemistry, Seoul National University, Seoul, 151-742, Korea

**Keywords:** LC-MS/MS, Nectar protein, Nectarin, *Nicotiana attenuata*

## Abstract

**Background:**

Floral nectar (FN) contains not only energy-rich compounds to attract pollinators, but also defense chemicals and several proteins. However, proteomic analysis of FN has been hampered by the lack of publically available sequence information from nectar-producing plants. Here we used next-generation sequencing and advanced proteomics to profile FN proteins in the opportunistic outcrossing wild tobacco, *Nicotiana attenuata*.

**Results:**

We constructed a transcriptome database of *N. attenuata* and characterized its nectar proteome using LC-MS/MS. The FN proteins of *N. attenuata* included nectarins, sugar-cleaving enzymes (glucosidase, galactosidase, and xylosidase), RNases, pathogen-related proteins, and lipid transfer proteins. Natural variation in FN proteins of eleven *N. attenuata* accessions revealed a negative relationship between the accumulation of two abundant proteins, nectarin1b and nectarin5. In addition, microarray analysis of nectary tissues revealed that protein accumulation in FN is not simply correlated with the accumulation of transcripts encoding FN proteins and identified a group of genes that were specifically expressed in the nectary.

**Conclusions:**

Natural variation of identified FN proteins in the ecological model plant *N. attenuata* suggests that nectar chemistry may have a complex function in plant-pollinator-microbe interactions.

## Background

Floral nectar (FN) is produced in many flowers to promote outcrossing mediated by pollinator visitation [1]. FN consists largely of sugars including sucrose, glucose, and fructose, regarded as rewards for animal pollinators. FN also contains several proteins, which are called nectarins [2-6]. Although the presence of nectar proteins was first described nearly 80 years ago [7], only a limited number of proteins have been identified and characterized. Of these, many are likely not directly involved in attracting or repelling pollinators [8,9], but rather function in sugar metabolism as well as in defense against microorganism proliferation, largely through the production of strong oxidants, such as hydrogen peroxide (H_2_O_2_) [10]. However, the over-accumulation of H_2_O_2_ produced by FN proteins has been shown to affect pollination behavior and reduces nectar removal by insect floral visitors [11].

The best characterized proteins in FN are nectarins (NECs). Five NECs have been identified from an ornamental tobacco, *Nicotiana langsdorffii* x *Nicotiana sanderae*[7,9]. The NEC1 (nectarin1) protein was first classified as a germin-like protein [12], and it was further characterized as manganese-containing superoxide dismutase involved in producing H_2_O_2_[5]. NEC5, a flavin-containing berberine bridge enzyme-like protein, possesses glucose oxidase activity catalyzing the oxidation of D-glucose to D-gluconic acid and H_2_O_2_[4]. High levels of H_2_O_2_ generated by NEC1 and NEC5 are closely associated with antimicrobial activity in the nectar [7]. Consistent with their functional similarity, expression patterns of the *NEC1* and *NEC5* genes are also similar in the ornamental tobacco; both are highly expressed in nectary tissues just prior to anthesis [5,7,12]. The NEC3 protein, which has high sequence similarity to the antioxidant storage protein, dioscorin, possesses both monodehydroascorbate reductase and carbonic anhydrase activities [7], and contributes to the maintenance of pH and oxidative balance in the nectar.

Other types of FN proteins have also been identified from diverse plant species. The flowers of leek (*Allium porrum*) predominantly produces alliin lyase, which catalyzes the conversion of alliins to allicins, and a mannose-binding lectin [13]. A GDSL lipase was found in the FN of *Jacaranda minosifolia*[14]. This enzyme possesses lipolytic esterase activities and probably plays a role in the release of free fatty acids that may be involved in pollinator attraction. The GDSL lipase may also act against microbes by disrupting spore membrane integrity [15]. In addition, the antimicrobial proteins RNase, peroxidase, and endochitinase have been identified from the FN of *Petunia hybrida*[16]. Thus, several FN proteins likely function in a complicated multipronged defense against microbes in this otherwise palatable solution offered to reward pollinators.

Mass spectrometry has become a powerful analytical technique for protein identification in multicomponent samples spanning wide dynamic range of protein concentrations [17]. To decrease the complexity of the samples, proteins are separated by one- or two-dimensional (D) gel electrophoresis, visualized and digested with one of the proteolytic enzymes (typically trypsin) to produce peptides. The extracted peptides are separated by nano HPLC prior their injection into a tandem mass spectrometer where they undergo serial fragmentation by collision-induced dissociation (CID) to generate product ion spectra for as many peptides as possible.

The peptides are usually identified using database searching that relies on the correlation of the acquired peptide masses along with the corresponding masses generated *in-silico* from available genomic, EST, or protein sequence databases using dedicated software [18-20]. Although protein identification based on stringent database searching is highly specific and accurate, its application is limited to organisms with available sequence information or to homologous proteins from closely related species. Identification of protein families with poorly conserved domains, strong sequence polymorphisms, or from species phylogenetically distant from model organisms remains a major challenge for proteomic research. To increase the scope of studied organisms, an alternative strategy has been developed, that relies on *de novo* sequencing of the acquired MS/MS spectra followed by their searching using specialized sequence-similarity search engines in an error tolerant manner to identify peptides on the basis of their homology to peptides in the database [21-23].

Although some studies of nectar proteomes from non-model plants using similarity-based searching have been reported, there is no doubt that a genomic database of nectar-producing plants would greatly improve nectar protein identification [24]. In addition, the sequences of genes encoding nectar proteins enable an examination of the transcriptional regulation of these proteins. In this work, we conducted proteomic analysis using FN collected from *Nicotiana attenuata* accessions across the plant’s native habitat, the Great Basin Desert, Utah. *N. attenuata* flowers produce a relatively large volume of nectar to attract two main pollinators: *Manduca sexta* moths at night and hummingbirds in the morning [25-27]. We found natural variation in FN proteins among different *N*. *attenuata* accessions collected across the plant’s native habitat and identified FN proteins through stringent and homology-based database searching against public (NCBInr) and an in-house *N. attenuata* protein subdatabase.

## Results

### Variation of FN proteins among eleven *N. attenuata* accessions

We first characterized nectar volume and protein content in flowers of the *N. attenuata* Utah accession (UT), which was used to generate the 454-transcriptome database. Secretion of FN was developmentally regulated: nectar volume increased gradually during flower opening (Additional file 1A). The amount of proteins also increased with floral development (Additional file 1B). We therefore analyzed FN produced at late stages of nectary development (2nd-day open flowers) in the subsequent assays. Nectar volume of single flowers was 6.3 ± 1.6 μL/flower (mean ± SE, *n* = 12). The total amount of proteins per flower determined by Bradford assays was 388.72 ± 3.34 μg mL^-1^ (mean ± SE, *n* = 6).

To investigate the variation in nectar proteins among *N. attenuata* accessions, we collected the FN from 11 different accessions grown in a glasshouse and measured the FN volume and protein concentrations. The nectar volume was highly variable among accessions, ranging from 4 to 16 μL/flower (Additional file 2A). Among them, the Highway accession (HW) accumulated by far the largest volume of nectar (15.5 ± 2.8 μL/flower, *n* = 28) compared with other accessions. The protein content also varied between 60 and 480 μg mL^-1^ among the accessions (Additional file 2B). Interestingly, the protein content in FN of HW was significantly lower than other accessions. Generally, there was a weak negative correlation (Pearson correlation test: r = −0.62, *P* = 0.042) between nectar volume and protein content among accessions (Additional file 2C), suggesting that the total amount of proteins per flower are similar in *N. attenuata* accessions.

**Figure 1:**
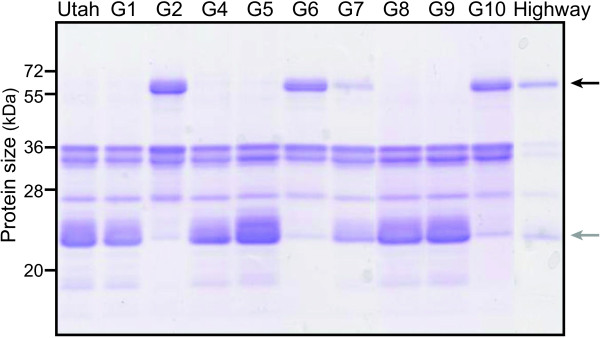
**1D gel electrophoresis of FN proteins from 11** ***N. attenuata *accessions.** Black and gray arrows indicate the most variable proteins among 11 different accessions. Several accessions (G2, G6, G7, G10, and Highway) which produced black-arrow indicated proteins (NECTARIN5) had less gray-arrow indicated proteins (NECTARIN1b) than other accessions (Utah, G1, G4, G5, G8, and G9) which had no black-arrow indicated proteins.

We next analyzed proteins in the FN collected from the 11 accessions by 1D gel electrophoresis (Figure 1). Nectar proteins were mainly distributed between 20 and 60 kDa. Interestingly, a negative correlation was observed between the amounts of two highly accumulated proteins among the accessions, indicated by black (NaNEC5, see below) or gray (NaNEC1b) arrows (Figure 1): several accessions (G2, G6, G7, G10, and HW) produced abundant amounts of NaNEC5 but less of NaNEC1b. Other accessions (UT, G1, G4, G5, G8, and G9) which produced no detectable amounts of NaNEC5 produced abundant amounts of NaNEC1b. In terms of protein content and nectar volume, HW differed most from the “control” UT accession. We therefore chose UT and HW for further FN protein analyses.

### Identification of FN proteins in Utah and highway accessions

To identify FN proteins in these two selected accessions of *N. attenuata*, we first separated FN proteins by 1D gel electrophoresis. Each gel from the UT and HW accession was divided into 15 fractions and analyzed by LC-MS/MS (Additional file 3A). Data analysis using stringent and similarity-based searching revealed that homologs of NEC1 and NEC3 proteins were enriched in FN of UT and HW, while the NEC5 homolog was only enriched in FN of HW (Additional files 4 and 5).

**Figure 2:**
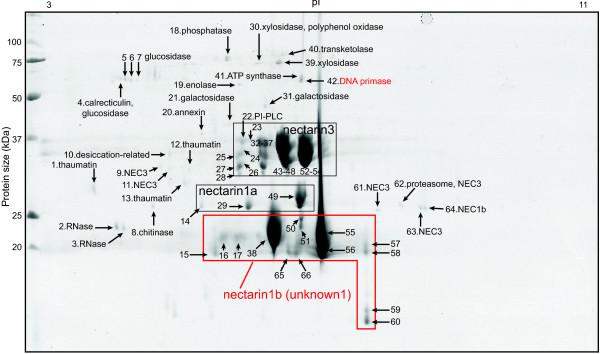
**Identification of FN proteins from *N. attenuata *Utah accession using LC-MS/MS analysis.** Floral nectar (FN) proteins from Utah accession were processed and analyzed as descripted in Additional file 17. Additional file 6 shows the list of identified proteins. Proteins in a red-box were identified in an in-house database not in NCBInr. NEC, nectarin.

**Figure 3:**
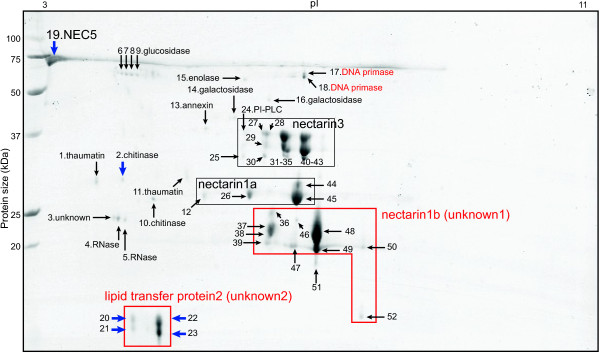
**Identification of FN proteins from *N. attenuata *Highway accession using LC-MS/MS analysis.** Floral nectar (FN) proteins from Highway accession were processed and analyzed as descripted in Additional file 17. Additional file 7 shows the list of identified proteins. Proteins in red-boxes were identified in in-house database not in NCBInr. Blue arrows indicate proteins found in Highway, not in Utah accession.

To increase the resolution of the analysis, we performed 2D gel electrophoresis of UT FN, which yielded 66 protein spots in the gel (Figure 2 and Additional file 6). Most proteins ranged in molecular mass from 20 to 40 kDa, consistent with the patterns observed in 1D gel electrophoresis. The most abundant proteins between 25 and 30 kDa were homologs (NaNEC1a) of NEC1 and germin-like proteins, and those between 30 and 37 kDa were homologs (NaNEC3) of NEC3 and carbonic anhydrase (Figure 2). Some abundant proteins below 25 kDa remained unassigned by searching against the public database (NCBInr) (unknown1 in the red box in Figures 2 and 3). However, we were able to identify these proteins in UT and also in HW accessions by searching against the *N. attenuata* protein subdatabase. They corresponded to the NaNEC1b (Na_454_00074) transcript encoding a germin-like protein (Additional files 6 and 7).

FN proteins of HW were mostly similar to those of UT, but several additional spots indicated by blue arrows were observed only in the HW accession (Figure 3 and Additional file 7). NaNEC5 proteins were detected only in HW FN as shown in the 1D gel analysis. The protein spot marked as ‵2′ was found only in HW FN and identified as a chitinase. Another HW-specific protein spots below 20 kDa (unknown2 in Figure 3) could not be identified using the public NCBInr database. Searching against the *N. attenuata* subdatabase resulted in a lipid transfer protein2 (Na_454_10170) (Additional file 7).

A number of other, heterogeneous proteins were also found in the FN of both accessions (Figures 2 and 3). These included pathogenesis-related (PR) proteins (thaumatins, calrecticulins, and chitinases), and defense-related proteins (annexins and RNases). Proteins involved in sugar metabolism, such as xylosidase, glucosidases, and galactosidases, were also identified. In addition, we found a number of additional enzymes, including phosphatase, enolase, ATP synthase, peptidase, phospholipase, and proteosome. The protein spots marked as ‵42′ in UT and marked as ‵17′, ‵18′ in HW corresponded to the Na_454_09241 transcript encoding a DNA primase where searching against an in-house database (Additional files 6 and 7). These results demonstrate the diversity of the FN proteome.

However, an alternative hypothesis to explain the diversity of the FN proteome could be that FN was contaminated with proteins from other floral tissues during nectar collection. To test this possibility, we first examined the reproducibility of our method. In 1D gel electrophoresis analysis, we were able to identify low abundant proteins, such as calrecticulins, chitinases, galactosidase, glucosidase, RNase, annexin, and enolase (Additional files 4 and 5). In addition, 2D gel electrophoresis of UT FN and HW FN were repeated three and two times, respectively, using independently collected nectars (Additional file 8). The majority of the low abundant spots in UT FN (Figure 2) and HW FN (Figure 3) were detected in the second or third replicates (Additional file 8). We also searched for fragments of ribulose bisphosphate carboxylase (RUBISCO) enzyme, which was found as a collection-related contamination of FN in our previous study [11]. No peptide fragment from RUBISCO proteins was detected in FN after modifying a collection method (See Methods). These data suggest that most of proteins identified in this study were FN proteins and free from contamination. However, some spots marked as ‵12′ and‵40-42′ in UT (Figure 2) and as ‵2′ in HW (Figure 3) appeared only once amongst the replicates, suggesting that they could be minor contaminants from other floral tissues (Additional file 8).

### NEC1 orthologs in *N. attenuata*

**Figure 4:**
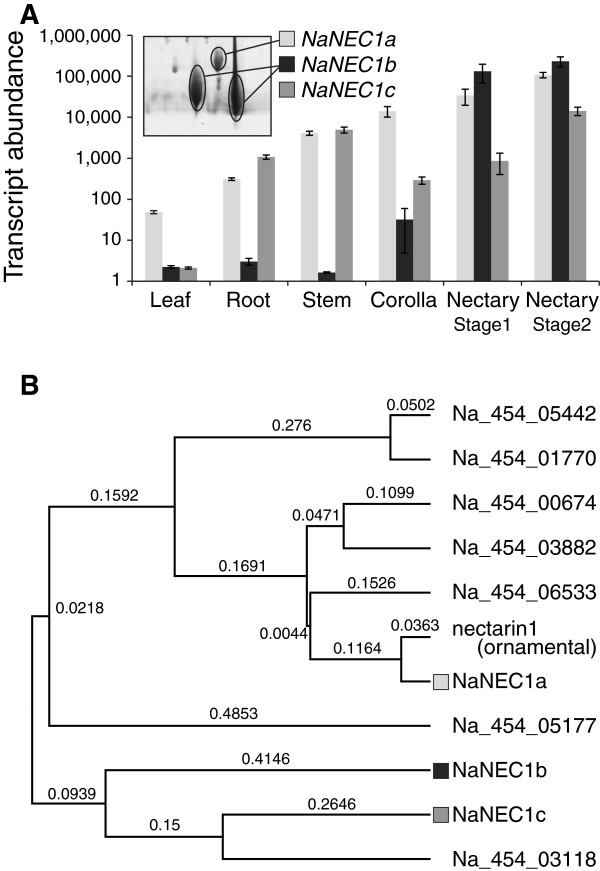
**Tissue-specific transcript abundance and phylogenetic analysis of *N. attenuata *nectarin1-like genes. (A)** The transcript levels of three nectarin1-like genes in leaf, root, stem, corolla, and nectary of *N. attenuata*. The mean intensities (±SE) of *NaNEC1a*, *b*, *c* genes are plotted on the y-axis in a logarithmic scale. Nectary was divided into two stages on the base of its maturation and color: early white nectary (stage1) and late pink nectary (stage2). **(B)** Phylogenetic relationship among 10 germine-like proteins in *N. attenuata* (Na) and NECTARIN1 from the ornamental tobacco (*Nicotiana langsdorffii* x *Nicotiana sanderae*). Putative full-length amino acid sequences were aligned and phylogenetic tree was constructed by the UPGMA (unweighted pair group method with arithmetic mean) method. The numbers given for each branch reflect the numbers of amino acid substitutions per site. NEC, nectarin.

Although the NEC1 protein is a major player in the nectar redox cycle of an ornamental tobacco [7], *N. attenuata* produced a relatively small amount of the NEC1 ortholog NaNEC1a, which is almost identical to the ornamental tobacco NEC1 (Additional file 9, Identities = 93%, Positives = 96%, Gaps = 0%). Instead, other germin-like proteins NaNEC1b (Na_454_00074) were more abundant in UT FN compared to NaNEC1a (Figure 4A, inset). Microarray expression data showed that the nectary-specific accumulation of *NaNEC1a* and *NaNEC1b* transcripts, but *NaNEC1b* transcript expressed more specifically than *NaNEC1a* in nectary (Figure 4A). Transcripts of an additional homologous gene, *NaNEC1c*, were abundant in the nectary (Figure 4A), but its corresponding protein was not detected in FN from either accession. The signal peptide [28] was predicted to be in the N-terminus of all three NaNEC1 isoforms, and C-terminal regions contained a superoxide dismutase domain, indicating functional conservation among the NaNEC1 isoforms (Additional file 9).

In addition to the three NaNEC1 proteins, seven germin-like proteins were found in a transcriptome database (Figure 4B), and their transcripts were found in various tissues (Additional file 10). To establish the phylogenetic relationships among the 10 germin-like proteins and the NEC1 of an ornamental tobacco, their amino acid sequences were analyzed using UPGMA (unweighted pair group method with arithmetic mean) with the Geneious software. The phylogenetic relationships were unrelated to the accumulation of each protein in FN. Proteins encoded by NaNEC1c shared a high similarity with NaNEC1b, but NaNEC1c were not found in the FN of either accession although the *NaNEC1c* transcripts accumulated to a similar level as *NaNEC1b* transcripts in nectary (Figure 4).

### NaNEC5s and lipid transfer proteins in *N. attenuata*

**Figure 5:**
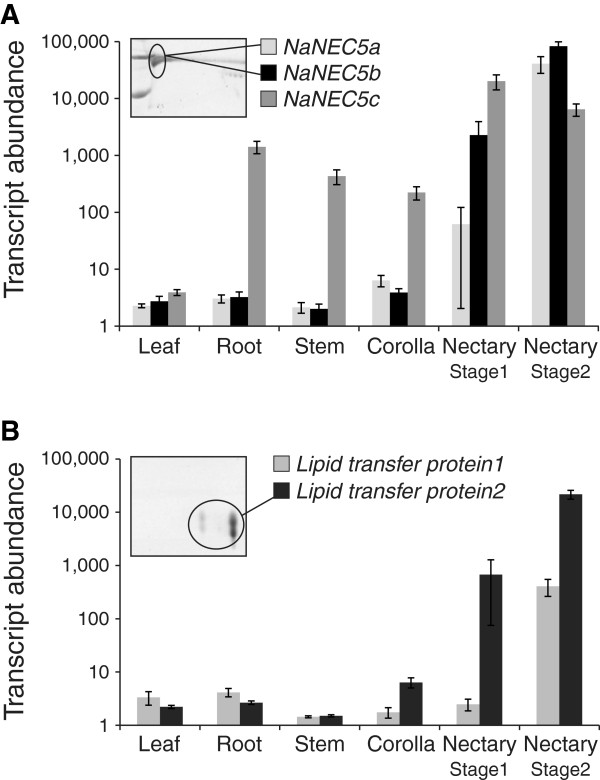
**Tissue-specific transcript expressions of NaNEC5s and lipid transfer proteins.** The transcript levels of three nectarin5-like genes **(A)** and two lipid transfer proteins **(B)** in leaf, root, stem, corolla, and nectary of *N. attenuata*. The mean intensities (±SE) of normalized expression for each gene are plotted on the y-axis in a logarithmic scale.

Interestingly, some of the *N. attenuata* accessions contained NaNEC5 proteins in their FN, and some did not (Figure 1). While homologs of NEC5 proteins were not detected in UT FN, three NEC5-like transcripts (*NaNEC5a*, *NaNEC5b*, and *NaNEC5c*) accumulated to high levels in UT nectaries (Figure 5A and Additional file 11). FN of HW contained two homologs of NEC5 proteins, namely NaNEC5a and NaNEC5b, among three NEC5-like proteins (Additional file 7). NaNEC5c protein was not detected in HW FN.

Similarly, the transcripts of two genes encoding a lipid transfer protein were highly abundant in UT nectary (Figure 5B), but neither was detected in 2D gel electrophoresis image of UT FN (Figure 2). However, lipid transfer protein2 was identified in HW FN (Figure 3 and Additional file 7).

### Microarray analysis of *N. attenuata* transcriptome in nectaries

**Figure 6:**
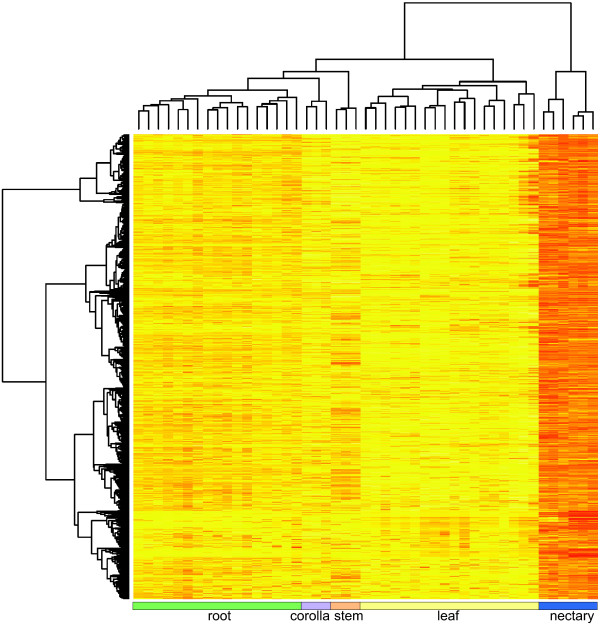
**Hierarchical clustering and heat map of nectary-specific genes in *N. attenuata*.** To identify nectary-specific genes in *N. attenuata*, a 44 K Agilent microarray designed for *N. attenuata* was used (GEO accession number GPL13527). Raw intensities were normalized using the 75th percentile value, and log_2_ transformation prior clustering analysis. After K-mean clustering, we selected a total of 3658 probe sets that expressed higher in nectary than in each other tissues; leaf, root, stem, and corolla. The R hclust function was used to generate the heat map and to carry out the hierarchical clustering based on the Euclidean distance. The accumulation levels of transcripts are color-coded: red, high expression; yellow, middle expression; white, low expression. All microarray data with each probe name were deposited in the NCBI GEO database (accession number GSE30287, GSE30124, GSE43394).

Physiological and chemical properties of FN are determined by the nectary tissues, which are responsible for nectar production and secretion [29]. We used a custom transcriptomic microarray to explore the accumulation of putative transcripts encoding FN proteins in the nectary compared to other tissues including leaves, roots, stems, and corollas. We also identified nectary-expressed genes by cluster analysis (Figure 6), and 8% of total transcripts (3658 probe sets) showed nectary-enriched expression. Among them we selected 198 transcripts, which passed the false-discovery rate threshold = 0% using the significance analysis of microarrays (SAM) algorithms in MulltiExperimental Viewer software [30] with significant (>10-fold) changes in accumulation between nectary and other plant tissues (Additional file 12). To increase the resolution of the analysis, we collected early and late developmental stages of nectary tissues (Additional file 13). Among the 198 nectary-specific transcripts, 4.5% of genes were preferentially expressed in early nectary development (stage1), and 29.8% were elevated in late nectary development (stage2). Transcripts of 65.7% of the genes accumulated to high levels in both developmental stages. Quantitative real-time PCR was conducted to validate microarray results (Additional file 14), and the resulting data revealed an excellent correspondence of microarray and qPCR results.

Most FN proteins are thought to play roles in the inhibition of microbial growth in sugar-rich nectar [24,31-33]. Therefore, we were particularly interested in FN proteins involved in this process. Several defense-related genes were highly accumulated in nectary (Additional file 12). However, some defense proteins known to be accumulated in FN of other species [16,34] were not detected in FN of *N*. *attenuata* by our analysis although their transcripts were abundant in the nectary. The transcripts of a floral defensin homolog [34] were abundant in the nectary (Na_454_02952), but its protein was not detected in FN. In addition nectary-specific PR (Na_454_06271) and RNase (Na_454_10706, Na_454_22815, Na_454_31748, Na_454_39811) were not detected in FN (Additional file 12). Interestingly, the accumulation of several transcripts encoding FN proteins: glucosidase (Na_454_00960), xylosidase (Na_454_00142), galactosidase (Na_454_03215), chitinase (Na_454_03840), and annexin (Na_454_00713), were reduced in the nectary compared to leaf, root, stem, and corolla tissues (Additional file 15). The data suggests that there is no strong correlation between transcript accumulation in nectary and its accumulation in FN.

We found a group of transcription factors (TFs) that were specifically expressed in nectary tissues. CRABS CLAW plays a critical role in nectary development of *A. thaliana*[35], and transcripts of a putative ortholog in *N. attenuata* (Na_454_06102, Additional file 16) were abundant in nectaries (Additional file 12). YABBY TF (Na_454_33877) and Class III HD-ZIP TF (Na_454_12695) also showed nectary-enriched accumulation patterns. Additionally, we found five nectary-specific MADS TFs which are putative homologs of SEEDSTICK TF involved in floral organ formation of *Arabidopsis* (Additional file 12) [36,37].

## Discussion

### Variations of FN proteins in *N. attenuata* accessions

In the nectar redox cycle proposed by Thornburg and colleagues (2004), NEC1 and NEC5 enzymes produce H_2_O_2_ in nectar, and both are highly expressed in an ornamental tobacco [7]. NaNEC1a and NaNEC1b proteins showed high sequence similarity to the ornamental tobacco NEC1, and was expressed in similar amounts per volume of nectar in the 11 accessions that we analyzed. Interestingly, *N. attenuata* has two NEC1-like proteins (NaNEC1a and NaNEC1b) in its FN, and accessions producing large amounts of NaNEC1b produce little or no NaNEC5 (Figure 1). NaNEC1b may serve the same function as NaNEC5 in hydrogen peroxide production in FN [7].

The FN of the HW accession of *N. attenuata* additionally contained NaNEC5a and NaNEC5b, and a lipid transfer protein compared to the UT FN. Interestingly, at least three *NaNEC5* genes and two genes encoding lipid transfer proteins were highly expressed in UT nectary, but the proteins were not detected in the UT FN. The most likely explanation is natural variation due to recent mutation in the secretion mechanism for NaNEC5 and lipid transfer protein. However, we cannot rule out the possibility of translation inhibition of these genes in the UT nectary. An alternative hypothesis posed at the functional level of analysis to explain *NaNEC5* expression in the UT nectary is that NaNEC5 may play an important role in nectary defense, not in the protection of FN.

Nectaries of *Nicotiana* species come into contact with the atmosphere and with precipitation, and contain both sugars and stomata, thus providing both food for pathogens and a means of entry into the plant [38,39]. Nectary defense is essential for resistance against microbe infection of the entire plant. In this work, we found that transcripts of nectarins, PRs, and defensins accumulated specifically in the nectary, but their proteins were not secreted to the FN, indicating that the nectary produces two groups of defense proteins: one for nectary defense, and another to provide axenic nectar.

### Nectary-specific transcriptome

The molecular mechanism regulating nectary development and nectar secretion still remains unclear [24]. Few genes involved in these processes have been characterized so far: CRABS CLAW TF in *Arabidopsis*[35] and MYB305 TF in tobacco [40] regulate nectary maturation, and mutation of *CELL WALL INVERTASE 4* transcript in *Arabidopsis*[41] alters nectar secretion. We were able to identify nectary-specific Na_454_06102 and Na_454_08038 transcripts, which are homologous genes of *CRABS CLAW* and *CELL WALL INVERTASE 4*, respectively (Additional file 12). In a previous study, silencing *MYB305*-like genes in *N. attenuata* results in premature termination of flower development [42]. In addition, large-scale transcriptome analyses in *Arabidopsis*[43] and *Brassica rapa*[44] can identify nectary-enriched gene family. Among them, lipid transfer protein and MATE transporter family were also highly accumulated in *N. attenuata* nectary. These data suggest that molecular components involved in nectary development are well-conserved in nectar-producing plants.

### Putative functions of FN proteins in *N. attenuata*

The most abundant FN proteins of *N. attenuata* were nectarins, which have been found in an ornamental tobacco and play a role in the maintenance of high levels of hydrogen peroxide in FN. In addition, PR proteins (chitinase, thaumatine), lipid transfer protein, and RNase were identified in the FN of *N. attenuata*. These proteins are also expected to help maintain an axenic FN environment which prevents microbial growth [7,31], but their exact functions have not yet been examined under natural conditions. Field experiments with plants silenced in the expression of or overexpressing putative nectar defense proteins would illuminate the roles of these putative nectar defense proteins and their impact on plants’ interactions with natural floral visitors. For instance, although increased peroxidase activity might better defend nectar against unwanted microorganisms, it would likely reduce pollinator visitation under natural conditions [11].

## Conclusions

The identification of FN proteins and their transcripts is difficult, largely due to the lack of sequence information from nectar-producing plants. A major model plant, *Arabidopsis* has nectary tissues producing nectar and valuable tools to dissect the molecular mechanisms in nectary development [29], but the nectaries are miniscule and only secret nectar in trace amounts, which makes nectar collection and analysis a challenge [24]. This study clearly shows that transcriptome sequence information combined with advanced LC-MS/MS analysis of gel separated proteins from a suitable model plant can be used to precisely identify FN proteins. In addition, natural variation in *N. attenuata* suggests a complex role of FN proteins in plant-pollinator-microbe interactions.

## Methods

### Workflow for protein identification in floral nectar

A workflow used for the identification of FN proteins in *N. attenuata* and their corresponding encoding genes, is depicted in Additional file 17. FN proteins were separated by 1 or 2D gel electrophoresis, and Coomassie-stained protein bands/spots were excised from the gel matrix and tryptically digested. Protein digests were then analyzed by nano-ultra-performance liquid chromatography-tandem mass spectrometry (LC-MS/MS). As *N. attenuata* is a non-model organism we used a combined approach for protein identification: highly specific stringent database searching using MASCOT software to identify conserved protein families and cross-species protein identification based on *de novo* interpretation of peptide tandem mass spectra followed by homology-driven MS BLAST searching to match proteins from phylogenetically related species. The de novo MS/MS sequence assemblies were searched against the NCBInr database and *N. attenuata* protein sequence database constructed by translating an in-house tanscriptome database [45]. Furthermore, we used a 44 K custom *N. attenuata* microarray [45] to examine the expression of transcripts encoding FN proteins in various plant tissues including leaves, stems, roots, corollas, and nectaries. Nectary tissues were analyzed at two stages based on their maturation and color: early development white nectary (stage1) and late development orange nectary (stage2) (Additional file 13).

### Plant material

Wild-type (WT) plants, *Nicotiana attenuata* Utah accession (30th inbred generation) and Highway accession (5th inbred generation) were grown from seeds originating from a natural population in Utah. G1, G2, G4, G5, G6, G7, G8, G9 and G10 accessions were collected in Utah [46]. Seeds were sterilized and germinated on Gamborg’s B5 medium as previously described [47]. Ten-day old seedlings were transferred to small pots (TEKU JP 3050 104 pots, Pöppelmann GmbH & Co. KG, Lohne, Germany) with Klasmann plug soil (Klasmann-Deilmann GmbH, Geesten, Germany) and after 10 days, seedlings were transferred to 1 L pots. Plants were watered by a flood irrigation system and grown in the glasshouse at 26 - 28°C under 16 h supplemental light from Master Sun-T PIA Agro 400 or Master Sun-T PIA Plus 600 W Na lights (Philips, Turnhout, Belgium) and 8 h dark conditions.

### Collection of FN and nectaries

Fully-opened flowers from *N. attenuata* accessions grown in a glasshouse were used for nectar analysis. Approximately 20 ~ 40 flowers on 10 individual plants for 1D-gel analysis and 200 ~ 300 flowers on 30 individual plants for 2D-gel analysis were carefully detached from flower stalks, and nectar was collected from the detached fully-opened flowers using a Pasteur pipette with a capillary tip. We measured nectar volume by inserting a calibrated 25 μL glass capillary into the detached corolla tube.

Development of the *N. attenuata* nectary is similar to the development of an ornamental tobacco (*N. langsdorffii* x *N. sanderae*) nectary [10]. To examine transcriptome changes during nectary development, nectary tissues were roughly divided into two developmental stages: small, white, early-stage nectaries (stage 1) and large, orange, mature nectaries (stage 2) which ripen during flower development. Early and late developmental nectaries from approximately 600 flowers (200 flowers per day) were collected between 8 to 10 p.m. from the UT accession grown in the glasshouse to isolate total RNA.

### Sequence alignment and phylogenetic analysis

Putative full-length sequences of nectarin1a (NCBI accession number JX871361), nectarin1b (accession number JX871362), nectarin1c (accession number JX871363), nectarin5a (accession number JX871364), nectarin5b (accession number JX871365), nectarin5c (accession number JX871366), lipid transfer protein1 (Na_454_00582, accession number JX871367) and lipid transfer protein2 (Na_454_10170, accession number JX871368) were deduced from a *N. attenuata* 454-transcriptome sequence database.

The amino acid sequences were aligned using the Geneious program V5.3 (http://www.geneious.com). The numbers of amino acid substitutions were estimated by a Jukes-Cantor model using a BLOSUM 62 matrix, through a global alignment with free end gaps option. A phylogenetic tree was reconstructed by the Unweighted Pair Group Method with Arithmetic Mean (UPGMA) method. These analyses were performed using Geneious software V5.3 (http://www.geneious.com).

### Microarray data analysis

Three biological replicates of each plant tissues were used for microarray. Total RNA isolation, hybridization, and data processing were carried out according to the procedures described in [45] All microarray data with each probe name were deposited in the NCBI GEO database (accession number GSE30287 for leaf and root [45], GSE30124 for stem, GSE33682 for corolla [48], GSE43394 for nectary).

MultiExperiment Viewer (MeV) software [49] was used for k-means clustering with the following parameter settings: Euclidean distance, number of clusters = 20 and for the significance analysis of microarrays analysis with the following parameter settings: false discovery rate = 0, number of permutations = 100. The R hclust function [50] was used to generate the heat map and to carry out the hierarchical clustering based on the Euclidean distance. To validate microarray results, quantitative real-time PCR was conducted using gene-specific primers (Additional file 18) as previously described in [51].

### 2D gel electrophoresis

Protein extracts were prepared from each accession (Utah and Highway) using 2-D Clean-up Kit (GE Healthcare). Sample aliquots containing 300 μg for Utah and 150 μg for Highway accession were subjected for 2D gel electrophoresis performed as described by Görg *et al.*[52]. Briefly, air dried pellets obtained from the protein extraction were solubilized in 420 μL of lysis buffer containing 8 M Urea, 4% Chaps, 0.5% pharmalyte (pH 3 to 11), 60 mM DTT. Particles were removed by spinning samples down for 1 min at 10000 rpm in a bench centrifuge. Sample aliquots were diluted in rehydration buffer (8 M Urea, 2% Chaps, 0.5% Pharmalytes (pH 3 to 11), 0.2% DTT, 0.002% bromophenol blue) to achieve a final volume of 500 μL. 24 cm IPG strips (ImmobilineTM DryStrips pH 3 to 11NL, GE Healthcare) were rehydrated overnight at 20°C. Isoelectric focusing was performed on an EttanTM IPGphor IITM system (GE Healthcare) at constant 75 μA per strip and constant temperature of 20°C using a following program: at 500 V h^-1^, at 1000 V h^-1^, and at 5000 V h^-1^. Proteins were then reduced by incubation of IPG strips with buffer containing 6 M Urea, 30% glycerol, 50 mM Tris–HCl pH 8.8, 2% SDS, and 1% DTT for 20 min and alkylated in buffer containing 6 M Urea, 30% glycerol, 50 mM Tris–HCl pH 8.8, 2% SDS, and 2.5% iodoacetamide (20 min in dark).

The IPG strips were sealed on 15% acrylamide-bisacrylamide gels with a solution containing 0.5% agarose in Tris SDS-PAGE buffer. Electrophoresis was performed at 40 mA/gel with a voltage limit of 500 V at 25°C. A solution containing 25 mM Tris–HCl (pH 8.8), 192 mM glycin, 0.1% SDS was used as a running buffer. After fixation of gels for one hour in a solvent containing 20% methanol and 1% of 85% o-phosphoric acid, they were stained overnight with Roti®Blue.

### In-gel digestion of samples for LC-MS/MS analysis

Protein bands of interest were cut from the gel matrix and in-gel digestion was carried out as described [53]. Proteins were reduced (10 mM dithiothretol, 1 h, 56°C) and alkylated (55 mM iodoacetamide, 45 min in the dark). Destained, washed, dehydrated gel pieces were rehydrated for 60 min in 0.5 μM solution of porcine trypsin in 25 mM ammonium bicarbonate buffer at 4°C and then digested overnight at 37°C. The tryptic peptides were extracted from gel pieces with extraction buffer (70% ACN/5% formic acid) and the extracts were dried down in a vacuum centrifuge. For LC-MS analysis samples were reconstructed in 10 μL aqueous 1% formic acid. Depending on staining intensity, 5 to 8 μL of sample were injected into LC-MS/MS system.

### LC-MS/MS analysis

A nanoAcquity nanoUPLC system (Waters, Manchester, UK) was used for peptide separation. Samples were injected onto a Symmetry C18 trap-column (20 × 0.18 mm, 5 μm particle size) using a mobile phase of 0.1% aqueous formic acid at a flow rate of 15 μL min^-1^ and separated on a nanoAcquity C18 column (200 mm × 75 μm ID, C18 BEH 130 material, 1.7 μm particle size) by in-line gradient elution at a flow rate of 0.350 μl min^-1^ using an increasing acetonitrile gradient from 1% to 95% B over 90 min (Buffers: A, 0.1% formic acid in water; B, 100% acetonitrile in 0.1% formic acid).

The eluted peptides were transferred into a Synapt HDMS Q-Tof tandem mass spectrometer equipped with a nanolockspray ion source (Waters, Manchester, UK). The mass spectrometer was operated in the data-dependent acquisition (DDA) mode using MassLynx software (version 4.1); the acquisition cycle consisted of a survey scan covering the range of *m*/*z* 400 to 1500 Da followed by MS/MS fragmentation of the four most intense precursor ions collected over a 1 sec interval in the range of *m*/*z* 50 to 1700 Da. A 650 fmol μL^-1^ human Glu-fibrinopeptide B in 0.1% formic acid/acetonitrile (1:1, v/v) was infused as the external mass calibrant at a flow rate of 0.5 μL min^-1^ through the reference NanoLockSpray source every 30 seconds.

### Data processing and protein identification

DDA raw files were processed using ProteinLynx Global Server Browser (PLGS) software (version 2.5, Waters, Manchester, UK) under baseline subtraction, smoothing, de-isotoping, and lockmass-correction. Pkl-files of MS/MS spectra were generated and searched using MASCOT software (version 2.3, installed on a local server) against NCBInr database (updated September, 11, 2011, containing 15270974 sequence entries) combined with *N. attenuata* protein subdatabase (containing 166315 sequences, constructed from in-house created EST database by its translation from all six reading frames). Mass tolerances for precursor and fragment ions were 15 ppm and 0.03 Da, respectively. Other search parameters were: instrument profile, ESI-Trap; fixed modification, carbamidomethyl (cysteine); variable modification, oxidation (methionone); up to 1 missed cleavage were allowed. Criteria to distinguish between confident and non-confident protein identifications were following: hits were considered as confident if at least three peptides were matched with ion scores above 25, or proteins were identified by one or two peptides with score of 50 or better.

In parallel acquired tandem mass spectra were subjected to homology-based database searching. Here, processed MS/MS spectra were first searched using PLGS software (using the same search parameters as described above) against a subdatabase containing common background proteins (human keratins and trypsin). Spectra remained unmatched by database searching were interpreted *de novo* using following sequencing parameters: fragment mass deviation, 0.002 Da, and ladder score (percentage of expected y- and b-ions) exceeding 40. Subsequently obtained *de novo* peptide sequences were subjected for homology-based database searching using MS BLAST program (Shevchenko *et al.*, 2001) installed on local server. MS BLAST searches were performed against complete NCBInr protein database (updated on August, 10, 2011, containing 14977208 sequence entries) as well as against *N. attenuata* (containing 166315 sequences, constructed in-house as described before). Statistical significance of hits was evaluated according to MS BLAST scoring scheme [23].

## Competing interests

The authors declare that they have no competing interests.

## Authors’ contributions

PS, DK, SK designed and conducted experiments, NW performed LC-MS/MS analysis and identified nectar proteins, PS, NW, ITB, and SK wrote the manuscript, AS, CP, ITB, and SK supervised the research. All authors approved the final manuscript.

## Supplementary Material

Additional file 1 **Floral nectar volumes and protein accumulations in *N. attenuata *Utah accession at different floral stages.** Mean (±SE) levels of floral nectar (FN) volumes (A) and 1D gel electrophoresis of FN proteins (B) at each floral stage. *N. attenuata* produces two different types of flowers. Morning-opening flowers (MoFs) open their corollas during the early morning (1st) and re-open during the next night (2nd). Night-opening flowers (NoF) open their corollas during the night (1st) and re-open during the next night (2nd). FN of the MoF and the NoF was harvested between 7 and 9 am. Click here for file

Additional file 2 **Floral nectar volumes and protein content among 11 different *N. attenuata *accessions.** Mean (±SE) levels of FN volumes (A) and protein amounts (B) of 11 different accessions. A slight negative correlation between FN volume and FN protein content among accessions was observed (C). FN was harvested between 7 and 9 am. Click here for file

Additional file 3 **Identification of floral nectar proteins separated by 1D gel electrophoresis.** (A) 1D gel electrophoresis of FN. FN was collected from Utah and Highway accession plants between 7 and 9 a.m. Individual bands were eluted and analyzed using nanoUPLC-MS/MS. (B) Major proteins identified in the individual bands (Additional files 4 and 5). NEC, nectarin. Click here for file

Additional file 4 Identified floral nectar proteins accumulated in Utah after 1D gel electrophoresis. Click here for file

Additional file 5 Identified floral nectar proteins accumulated in Highway after 1D gel electrophoresis. Click here for file

Additional file 6 List of floral nectar proteins identified in Utah after 2D gel electrophoresis. Click here for file

Additional file 7 List of floral nectar proteins identified in Highway after 2D gel electrophoresis. Click here for file

Additional file 8 **2D gel electrophoresis of FN proteins from *N. attenuata *Utah and Highway genotypes.** We independently collected FN from approximately 200 flowers in (A) Utah and (B) Highway genotype for each electrophoresis. Click here for file

Additional file 9 **Protein alignment of nectarin1 orthologs in *N. attenuata*.** Full-length amino acid sequences were aligned using the Geneious software. Red-bars indicate signal peptide sequences [28]. NEC, nectarin. Click here for file

Additional file 10 **Tissue-specific transcript abundances encoding germin-like proteins in *N. attenuata*.** Click here for file

Additional file 11 **Protein alignment of nectarin5 orthologs in *N. attenuata*.** Full-length amino acid sequences were aligned using the Geneious software. NEC, nectarin. Click here for file

Additional file 12 **Nectary-specific transcriptome in *N. attenuata*.** A 44 K Agilent microarray designed for *N. attenuata* was used (GEO accession number GPL13527) to identify nectary-specific genes. Raw intensities were normalized using the 75th percentile value and log_2_ prior clustering analysis. We selected a total of 198 transcripts that expressed 10 times higher in nectary tissue than in each other tissues; leaf, root, stem, and corolla. Click here for file

Additional file 13 **Development of *N. attenuata*’s floral nectary.** Click here for file

Additional file 14 **Quantitative real-time PCR to validate microarray data.** The transcript levels of (A) NaNEC1b, (B) NaNEC5a, (C) Lipid transfer protein1, (D) Na_454_00830 (Amino acid transporter), and (E) Na_454_21830 (MATE) in leaf, stem, corolla, and nectary of *N. attenuata*. The mean intensities (±SE) of two technical replicates are plotted on the y-axis in a logarithmic scale. Nectary was divided into two stages on the base of its maturation and color: early white nectary (stage1) and late pink nectary (stage2). Click here for file

Additional file 15 Tissue specific expressions of transcripts encoding FN proteins accumulated in Utah. Click here for file

Additional file 16 **Protein alignment of a CRABS CLAW ortholog in *N. attenuata*.** Full-length amino acid sequences were aligned using the Geneious software. TAIR accession number of CRABS CLAW (CRC) is At1g69180. Click here for file

Additional file 17 **Experimental workflow used for identification of floral nectar proteins and their encoding genes in *Nicotiana attenuata*.** Floral nectar samples were collected from *N. attenuata*. Proteins were extracted and separated by 1D and 2D gel electrophoresis, excised from the gel matrix, tryptically digested, and analyzed using LC-MS/MS. Acquired tandem mass spectra were processed using highly specific stringent (MASCOT software) and sequence-similarity database searching (*de novo*/MS BLAST). a) Processing of raw data under baseline subtraction, smoothing, deisotoping, lockmass-correction and generating pkl-files for Mascot database searching. b) The spectra were first searched against a subdatabase containing common contaminants (keratins, trypsin) in order to remove the corresponding MS/MS spectra from the raw-files before *de novo* sequencing. The remaining spectra were sequenced *de novo* and searched using MS BLAST to identify protein hits. c) The protein subdatabase of *N. attenuata* was created by translating assembled transcripts in all reading frames. A 44 K Agilent microarray designed for *N. attenuata* was used to examine the expression of transcripts encoding FN proteins and nectary-specific genes. Click here for file

Additional file 18 Primer sequences for quantitative real-time PCR. Click here for file
