# Supplementary figures and images for: Natural variation in floral nectar proteins of two Nicotiana attenuata accessions
Source: BMC Plant Biol. 2013 Jul 13;13:101. doi: 10.1186/1471-2229-13-101 (PMC3728157; doi:10.1186/1471-2229-13-101)

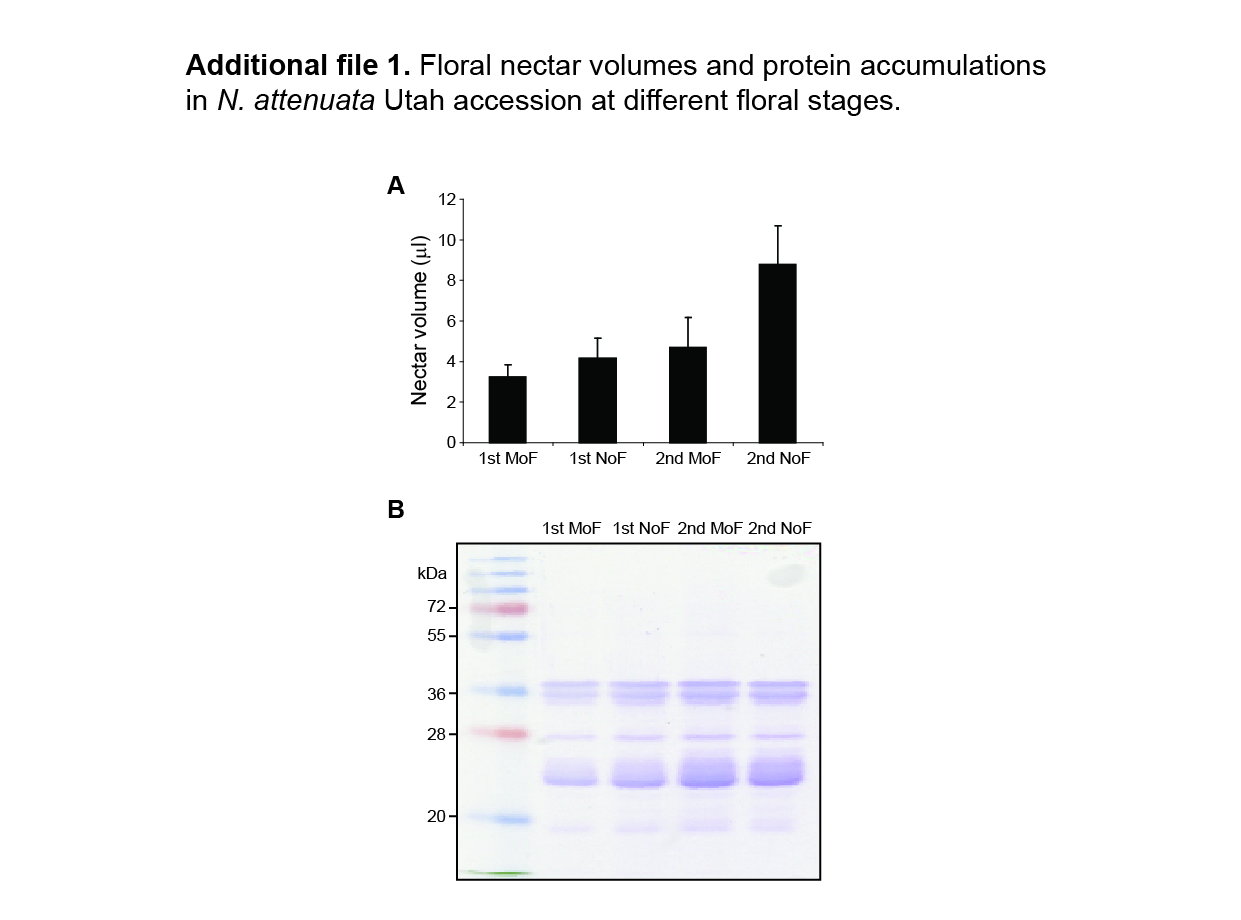

Supplement: Additional file 1 — Floral nectar volumes and protein accumulations in N. attenuata Utah accession at different floral stages. Mean (±SE) levels of floral nectar (FN) volumes (A) and 1D gel electrophoresis of FN proteins (B) at each floral stage. N. attenuata produces two different types of flowers. Morning-opening flowers (MoFs) open their corollas during the early morning (1st) and re-open during the next night (2nd). Night-opening flowers (NoF) open their corollas during the night (1st) and re-open during the next night (2nd). FN of the MoF and the NoF was harvested between 7 and 9 am. [file 1471-2229-13-101-S1.tiff]

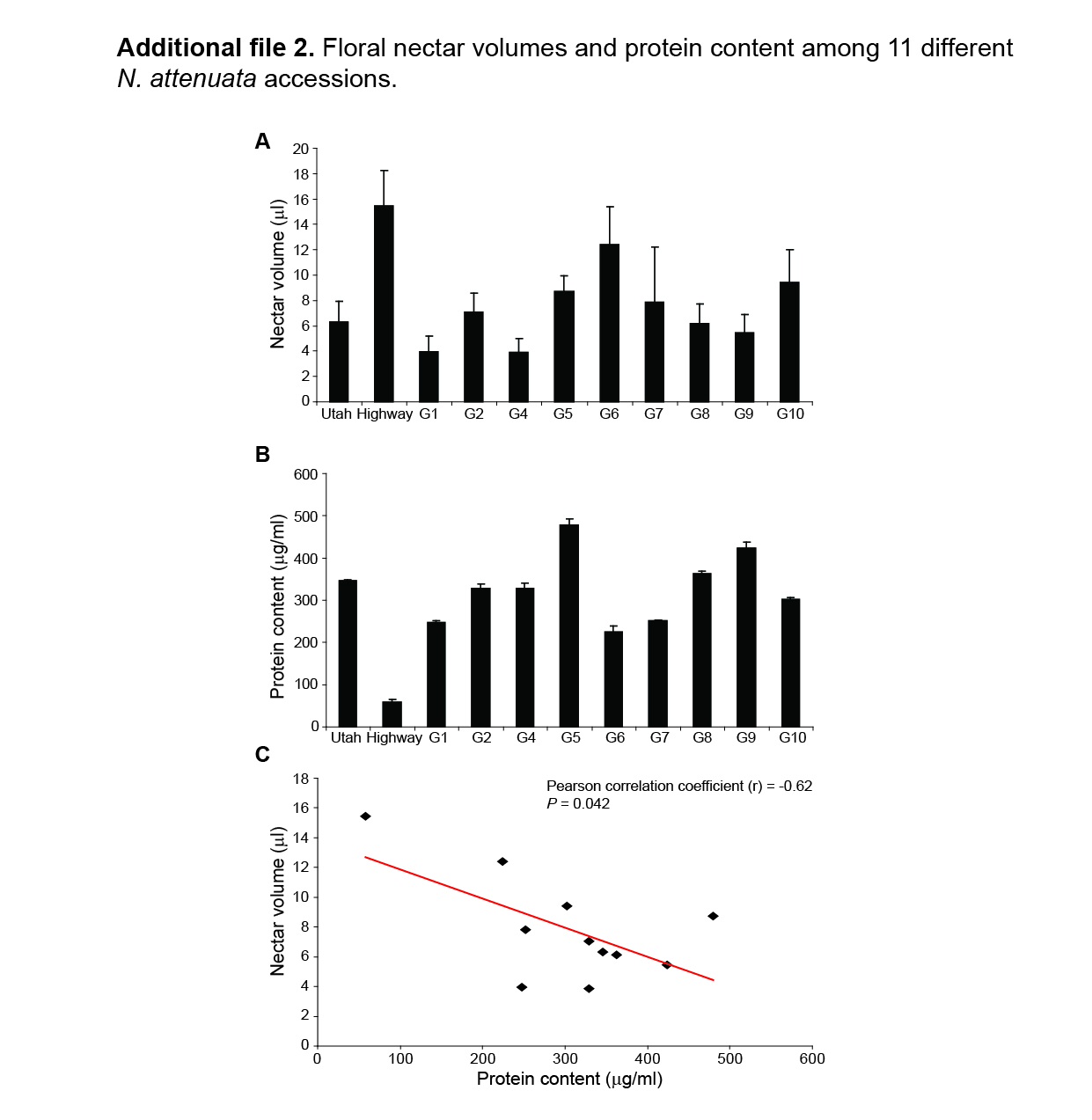

Supplement: Additional file 2 — Floral nectar volumes and protein content among 11 different N. attenuata accessions. Mean (±SE) levels of FN volumes (A) and protein amounts (B) of 11 different accessions. A slight negative correlation between FN volume and FN protein content among accessions was observed (C). FN was harvested between 7 and 9 am. [file 1471-2229-13-101-S2.tiff]

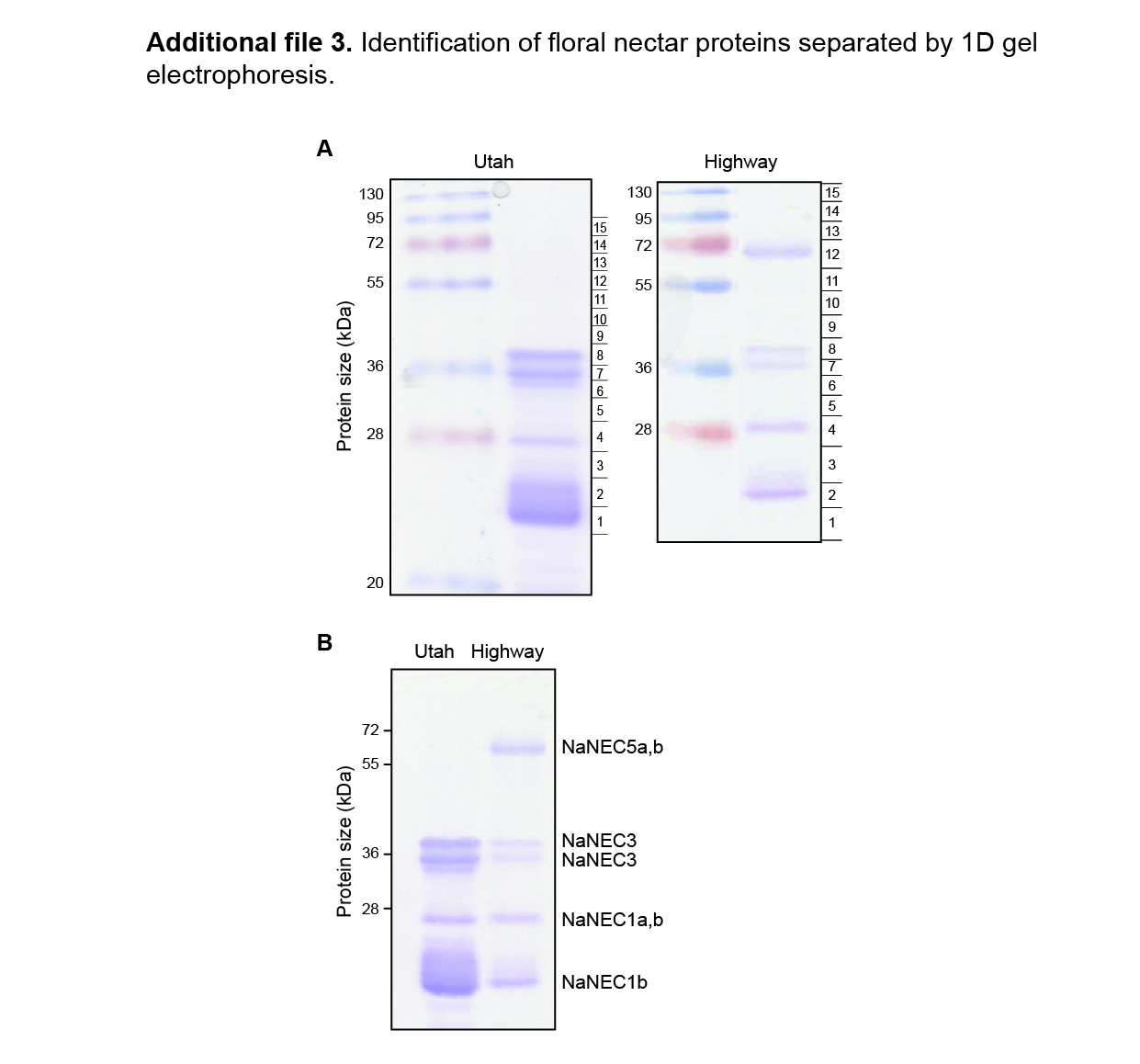

Supplement: Additional file 3 — Identification of floral nectar proteins separated by 1D gel electrophoresis. (A) 1D gel electrophoresis of FN. FN was collected from Utah and Highway accession plants between 7 and 9 a.m. Individual bands were eluted and analyzed using nanoUPLC-MS/MS. (B) Major proteins identified in the individual bands (Additional files 4 and 5). NEC, nectarin. [file 1471-2229-13-101-S3.tiff]

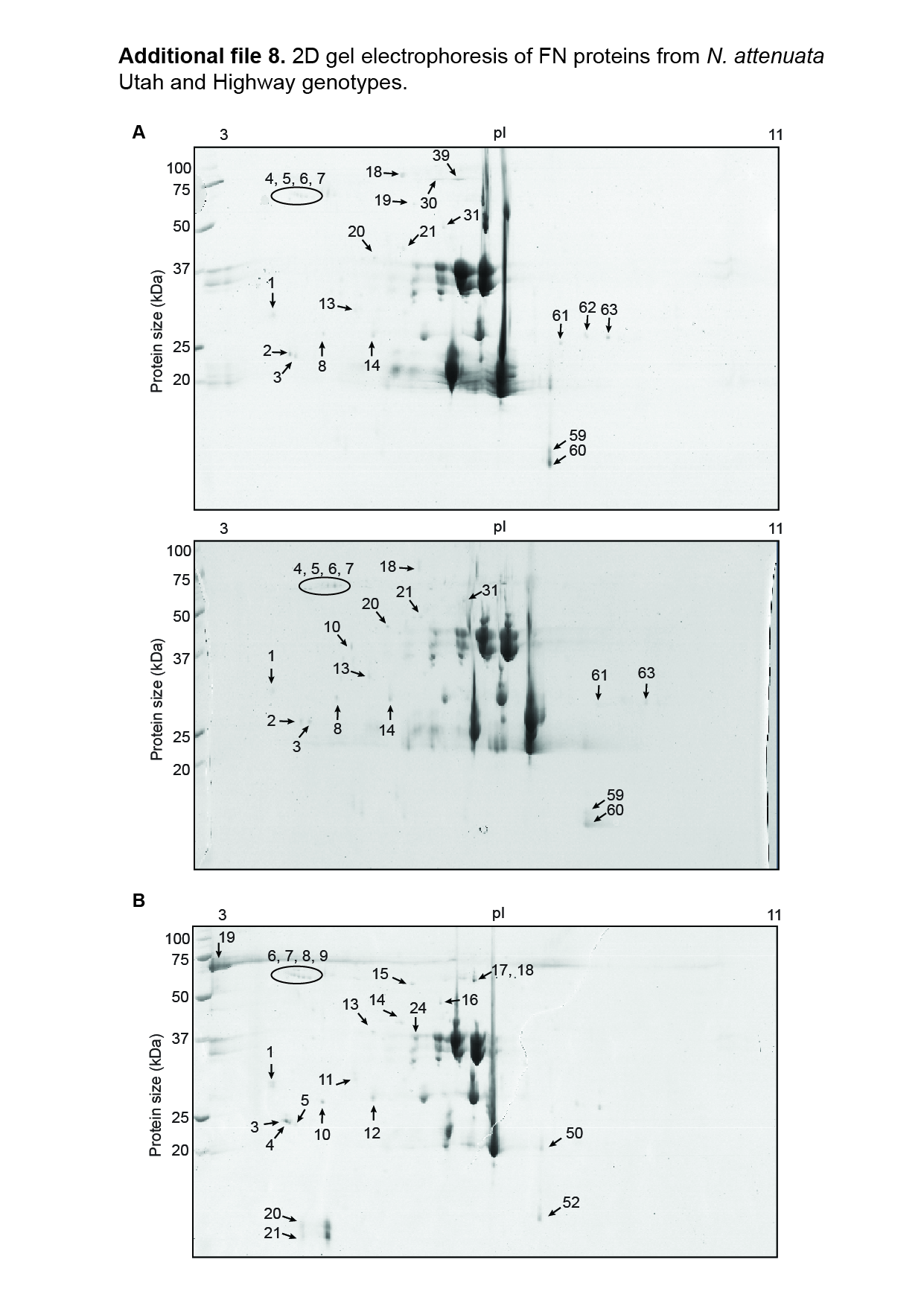

Supplement: Additional file 8 — 2D gel electrophoresis of FN proteins from N. attenuata Utah and Highway genotypes. We independently collected FN from approximately 200 flowers in (A) Utah and (B) Highway genotype for each electrophoresis. [file 1471-2229-13-101-S8.tiff]

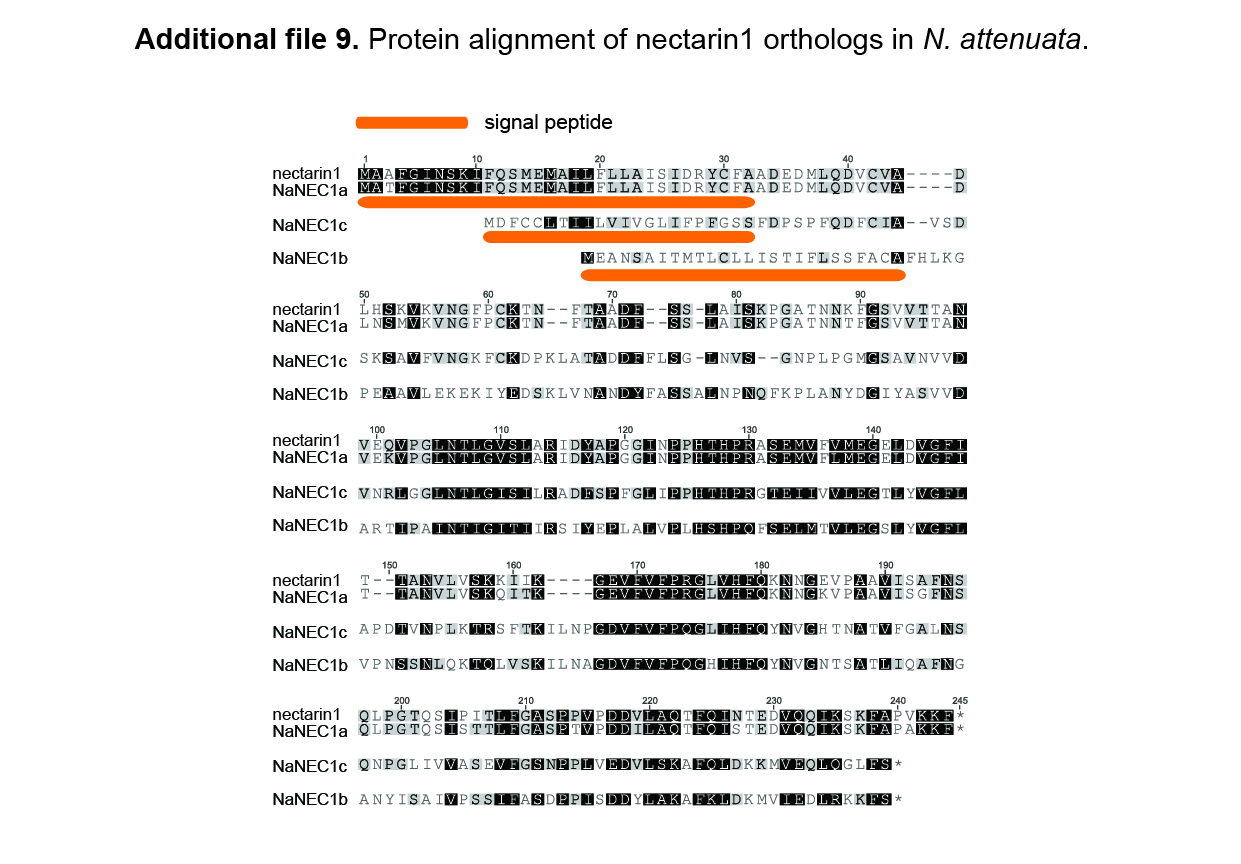

Supplement: Additional file 9 — Protein alignment of nectarin1 orthologs in N. attenuata. Full-length amino acid sequences were aligned using the Geneious software. Red-bars indicate signal peptide sequences [28]. NEC, nectarin. [file 1471-2229-13-101-S9.tiff]

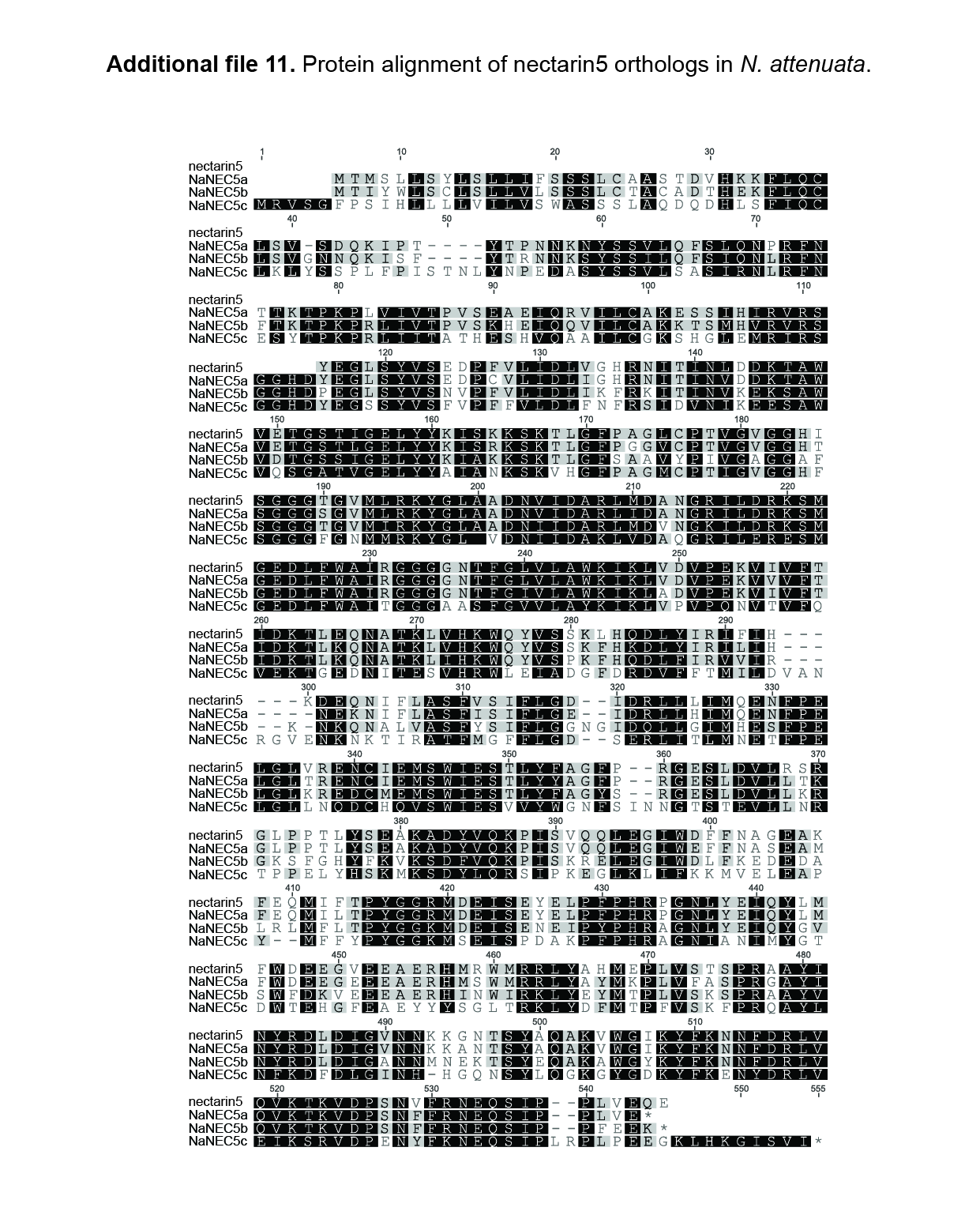

Supplement: Additional file 11 — Protein alignment of nectarin5 orthologs in N. attenuata. Full-length amino acid sequences were aligned using the Geneious software. NEC, nectarin. [file 1471-2229-13-101-S11.tiff]

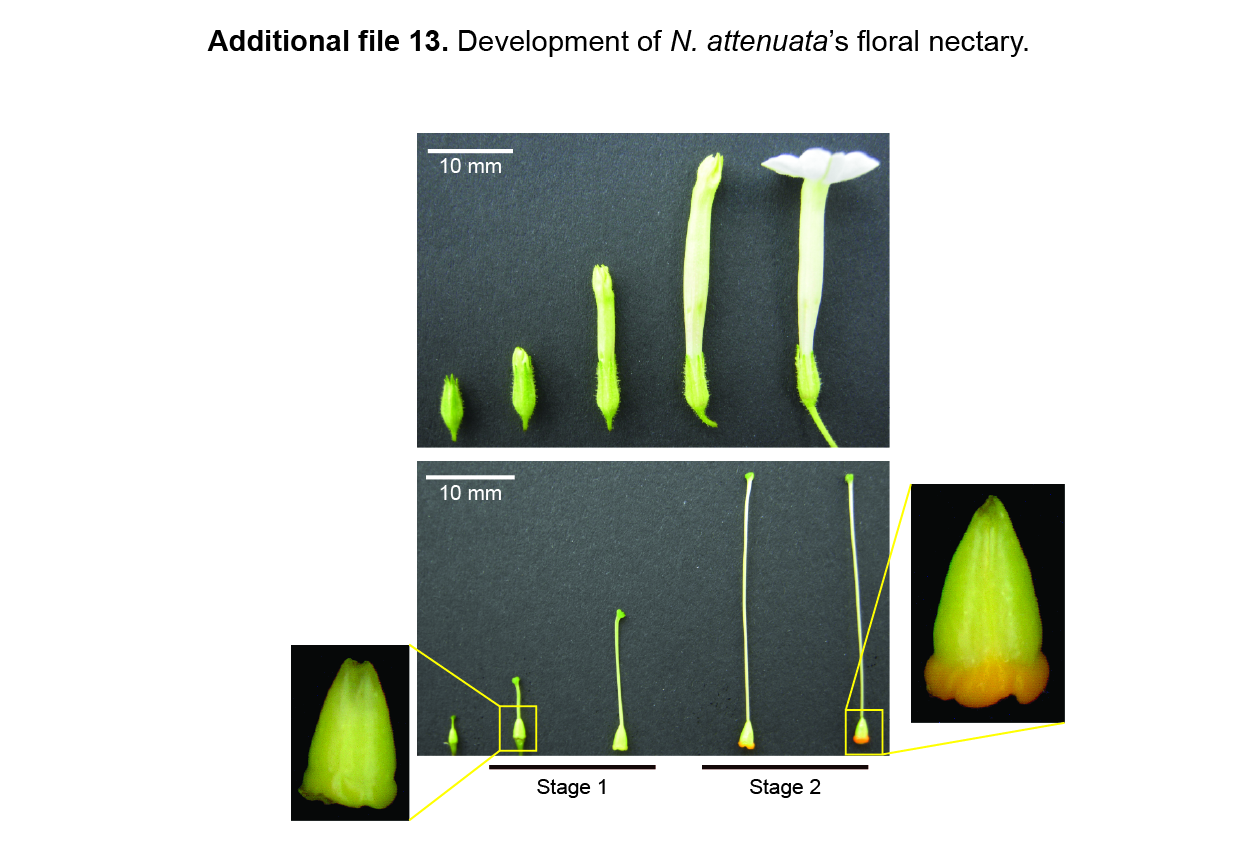

Supplement: Additional file 13 — Development of N. attenuata’s floral nectary. [file 1471-2229-13-101-S13.tiff]

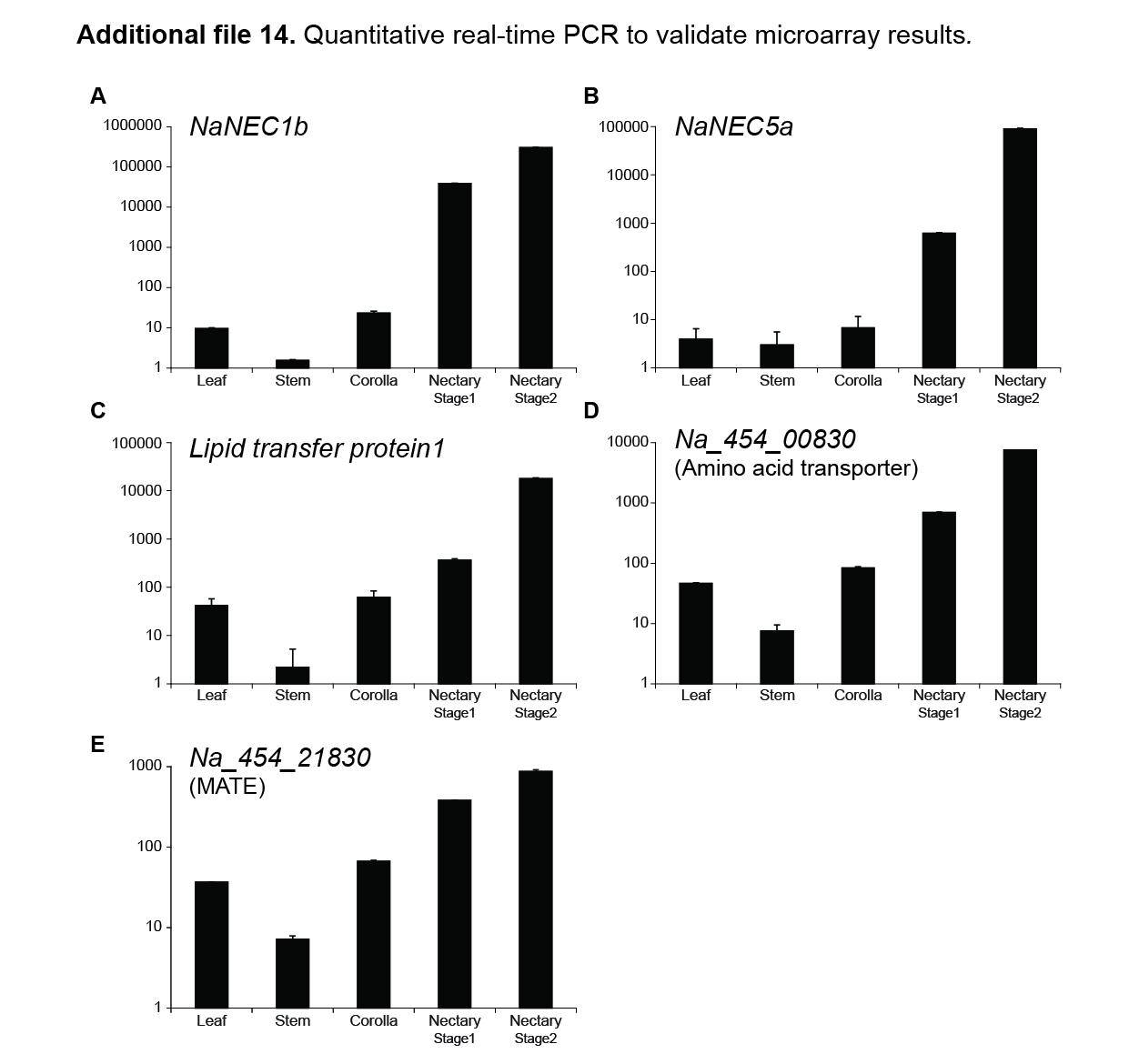

Supplement: Additional file 14 — Quantitative real-time PCR to validate microarray data. The transcript levels of (A) NaNEC1b, (B) NaNEC5a, (C) Lipid transfer protein1, (D) Na_454_00830 (Amino acid transporter), and (E) Na_454_21830 (MATE) in leaf, stem, corolla, and nectary of N. attenuata. The mean intensities (±SE) of two technical replicates are plotted on the y-axis in a logarithmic scale. Nectary was divided into two stages on the base of its maturation and color: early white nectary (stage1) and late pink nectary (stage2). [file 1471-2229-13-101-S14.tiff]

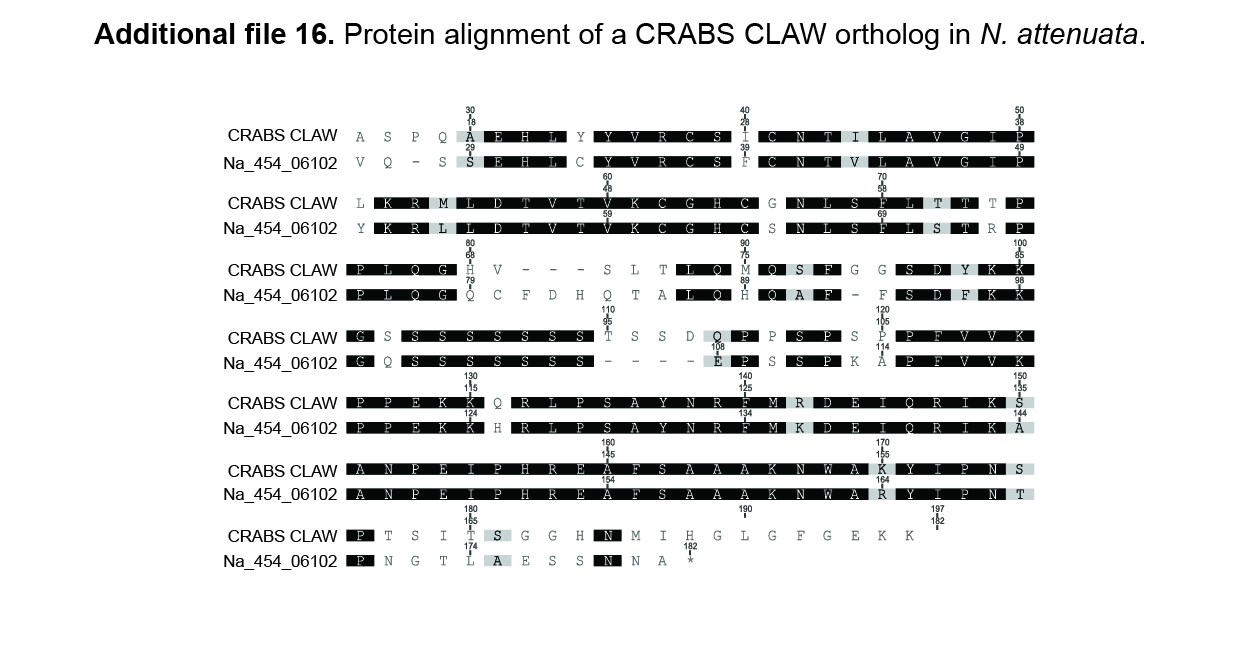

Supplement: Additional file 16 — Protein alignment of a CRABS CLAW ortholog in N. attenuata. Full-length amino acid sequences were aligned using the Geneious software. TAIR accession number of CRABS CLAW (CRC) is At1g69180. [file 1471-2229-13-101-S16.tiff]

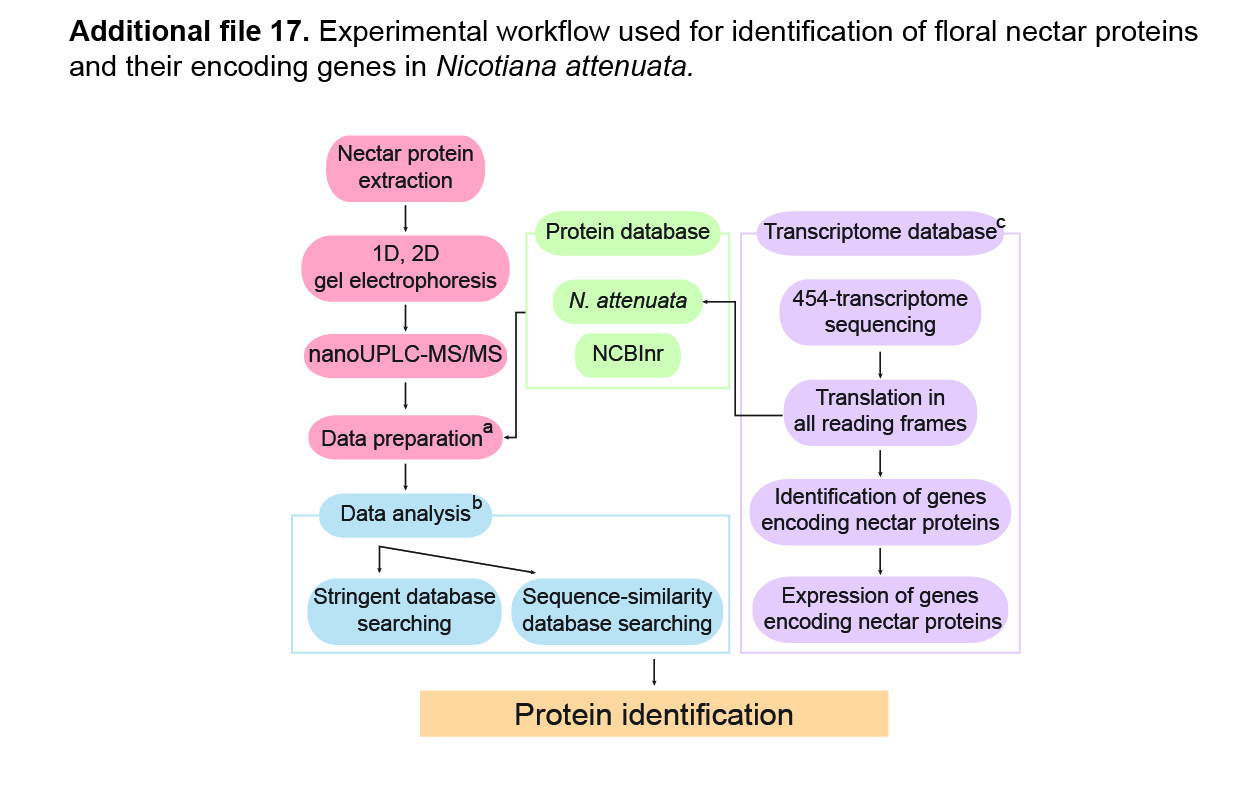

Supplement: Additional file 17 — Experimental workflow used for identification of floral nectar proteins and their encoding genes in Nicotiana attenuata. Floral nectar samples were collected from N. attenuata. Proteins were extracted and separated by 1D and 2D gel electrophoresis, excised from the gel matrix, tryptically digested, and analyzed using LC-MS/MS. Acquired tandem mass spectra were processed using highly specific stringent (MASCOT software) and sequence-similarity database searching (de novo/MS BLAST). a) Processing of raw data under baseline subtraction, smoothing, deisotoping, lockmass-correction and generating pkl-files for Mascot database searching. b) The spectra were first searched against a subdatabase containing common contaminants (keratins, trypsin) in order to remove the corresponding MS/MS spectra from the raw-files before de novo sequencing. The remaining spectra were sequenced de novo and searched using MS BLAST to identify protein hits. c) The protein subdatabase of N. attenuata was created by translating assembled transcripts in all reading frames. A 44 K Agilent microarray designed for N. attenuata was used to examine the expression of transcripts encoding FN proteins and nectary-specific genes. [file 1471-2229-13-101-S17.tiff]
